# Primary Care Patients’ Beliefs about Penicillin Allergy: Application of the Health Belief Model

**DOI:** 10.1007/s11606-025-10004-y

**Published:** 2025-11-14

**Authors:** Alysse G. Wurcel, Madelyn L. Eippert, Rachel A. Tam, Danielle C. Crabtree, Valerie E. Stone, Kimberly G. Blumenthal

**Affiliations:** 1 Sections of General Internal Medicine and Infectious Diseases, Boston Medical Center, Boston, MA USA; 2 Division of Pulmonary, Allergy and Sleep Medicine, Mayo Clinic, Jacksonville, FL USA; 3 The Mongan Institute, Massachusetts General Hospital, Boston, MA USA; 4 Department of Medicine, Massachusetts General Hospital, Boston, MA USA; 5 Harvard Medical School, Boston, MA USA; 6 Division of General Internal Medicine, Department of Medicine, Brigham and Women’s Hospital, Boston, MA USA

**Keywords:** beta-lactam, delabel, hypersensitivity, penicillin, qualitative

## INTRODUCTION

Interventions to support clinicians in the process of allergy reconciliation and verification are needed.^1^ Most people who report a penicillin allergy are not truly allergic.^1^ The “de-labeling” of an inaccurate penicillin allergy supports increased access to optimized antibiotic therapy.^1^ The American Board of Internal Medicine endorses the Choosing Wisely Campaign which promotes penicillin allergy de-labeling.^2^ Clinicians report concerns of perceived patient reluctance about addressing potentially inaccurate penicillin allergy labels.^3^ The goal of this study was to understand patients’ beliefs about their penicillin allergy and interest in penicillin allergy evaluation using the Health Belief Model framework.

## METHODS

In the parent study, we conducted in-depth interviews of people with penicillin allergy labels receiving primary care at three Boston hospitals (Massachusetts General Hospital, Brigham and Women’s Hospital, and Tufts Medical Center, *n* = 60 people). For this study, we analyzed 42 patient interviews with no history of penicillin allergy testing. We applied the Health Belief Model, a framework to understand perceptions of disease and motivations for changing risks for disease^4^ to four belief domains: (1) level of belief of allergy, (2) allergy reaction risk, (3) amenability to penicillin allergy testing, and (4) anticipated acceptance of taking penicillin provided testing disproved the allergy (Table 1). The patient’s allergy history description was reviewed by an allergy physician (KGB) to determine risk level. Categorization of qualitative data was iterative; an initial review of ten transcripts assessed by two coders formed preliminary groupings, followed by three rounds of transcript review and consensus discussion. The coding framework was finalized with tandem review of all 42 transcripts and visualized with a Sankey Diagram.

**Table 1:** Coding Framework Used to Classify Patient Interviews Into HBM Domains

**Domain**	**Interview guide prompt**	**Categories**	**Definition**	**Example quotes**
1: Level of belief in penicillin allergy	“On a scale of 1–10, with 1 being the least confident and 10 being the most confident, how confident are you that you are allergic to penicillin?”	High belief	Numerical rating > 5, or stated high belief without numerical rating	“10… Because I had a reaction, and it was definitely because of that ’cause it was nothing else that would’ve caused it at the time” “Well, I guess I would say eight. I’m pretty confident.”
		Low belief	Numerical rating ≤ 5, or stated not confident in allergy without numerical rating	“I would say between two and three. I think I’m probably not" “Maybe even lower, maybe two. Given that I’ve taken a lot of amoxicillin, I’ve taken, like I said, Augmentin. I’ve taken other drugs in the same class with no issue”
2: Risk of true penicillin allergy based on reaction history	“Tell me how you learned about your penicillin allergy.”	High risk	Description of penicillin allergy history reviewed and determined “high risk” by allergist (K.G.B)	“They gave it to me, and I took it and when I got home, I started not feeling well and I couldn’t see. I told my husband, ‘I can’t see,’ so according to them I lost consciousness” “My PCP gave me penicillin, and I had anaphylaxis.”
		Low risk	Description of allergy history risk reviewed and determined “low risk” by allergist (K.G.B)	“I broke out in the hives, so the doctor told my mother that I must be allergic to penicillin and not to ever take it again” “I was given penicillin for an infection in a tooth, and I broke out in red, little bumps all over. I guess it wasn’t really over my whole body, but from what I remember, I think it was my arms and stuff.”
3: Amenability to penicillin allergy testing	“Now that you know a little bit more about the penicillin allergy testing, would you be comfortable with the skin testing if you were higher risk?” “Would you be comfortable with an amoxicillin challenge if you were lower risk?” “Based on the testing options I stated above, which option would you prefer?”	Amenable to test	Participant is willing to be penicillin allergy tested	“Yes” “It sounds like a good idea. I would probably, if anything, would do the skin rather than taking it if I had a choice.”
		Not amenable to test	Participant is not willing to be penicillin allergy tested	“Makes me not want to do it…It just seems not fun. I’m not sure the ability to take penicillin is worth the extra stuff” “No, no, honestly no, I don’t want it. And my daughters also will not allow it.”
4: Anticipated acceptance of taking penicillin after negative allergy test	“If a medical professional did a test that showed you did not have a penicillin allergy, how would that make you feel?” “How would you feel about taking penicillins?”	Acceptance	Participant would take penicillin if they were found to be negative for the allergy	“If I don’t have an allergy, I would be wide open to taking it, absolutely” “I would take it…’cause then it would show I’m not allergic”
		Situational acceptance	Participant would only take penicillin if tested negative and only if necessary on a case-by-case basis. Participant is hesitant and cautious about taking penicillin	“Right away if need be? Probably not. Like I said, if it was a point where you really needed that antibiotic, then that would be something different” “Like I said, if it came to life or death, and that’s the only thing that would save me, but I think I have more serious problems than that.”
		Unsure	Participant’s willingness to take penicillin if tested negative is unclassifiable or they are unsure	“I don’t know. I do not know. It’s gonna take me a while to be trusting in the penicillin category.” “I would imagine it’s better than taking these antibiotics that are making our bugs resistant.”
		Rejection	Participant would not take penicillin if the penicillin allergy test showed that they do not have a true allergy	"I don’t dare to try anything because like I told you, my heart and all that" “I went through too much. Projectile vomiting, instantaneous reactions like that, heartbeat going erratic.”

## RESULTS

**Figure 1:**
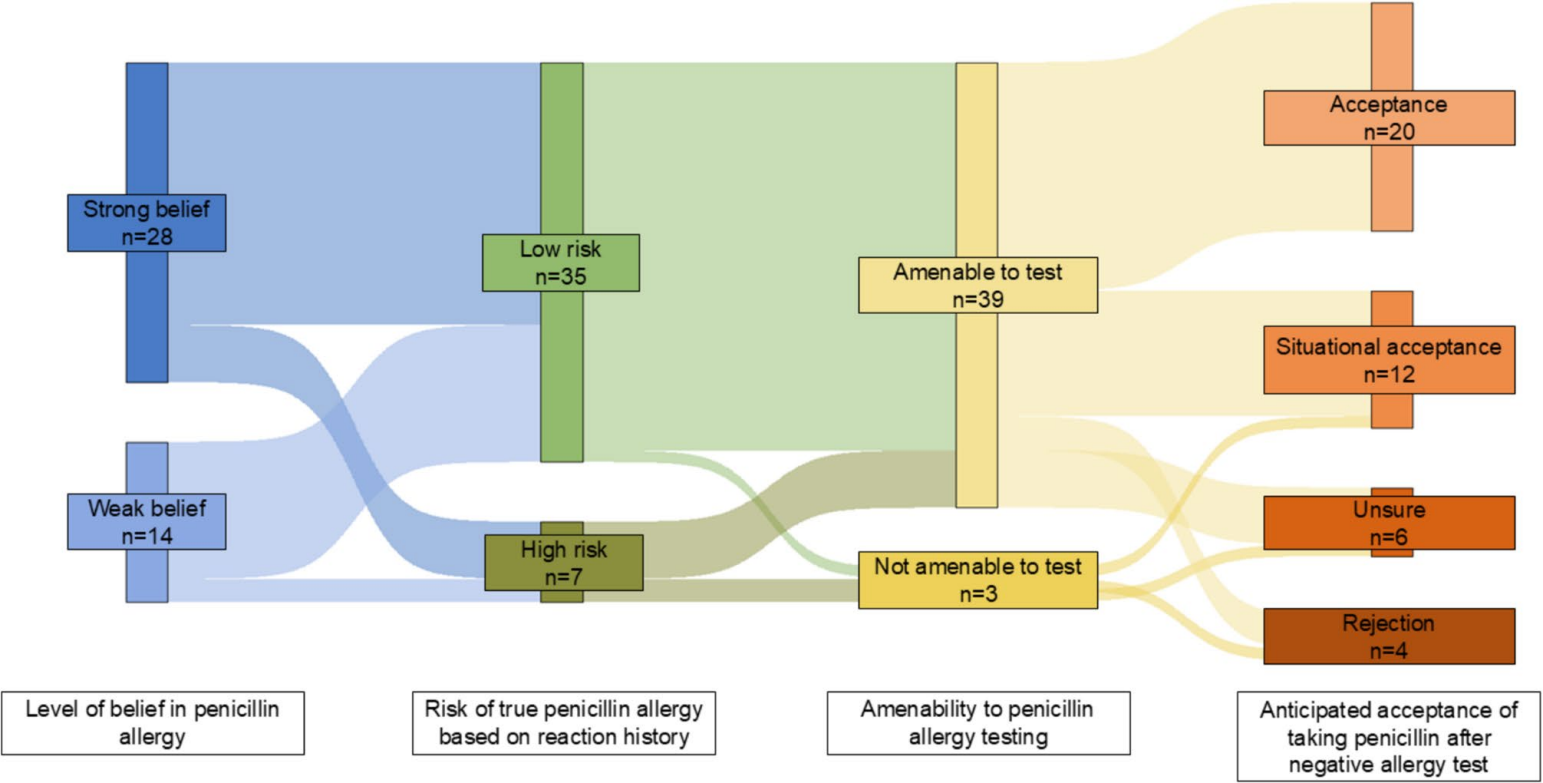
Sankey Diagram mapping patient penicillin allergy beliefs (*n* = 42) to the Health Belief Model. Each column of the diagram represents patient’s responses to interview questions. Individual pathways through the diagram included: weak belief-low risk-amenable to test-acceptance (*n* = 10, 24%), strong belief-low risk-amenable to test-acceptance (*n* = 9, 21%), strong belief-low risk-amenable to test-situational acceptance (*n* = 6, 14%), strong belief-low risk-amenable to test-unsure (*n* = 5, 12%), strong belief-low risk-not amenable to test-rejection (*n* = 2, 5%), strong belief-high risk-amenable to test-situational acceptance (*n* = 2, 5%), weak belief-low risk-amenable to test-situational acceptance (*n* = 2, 5%), strong belief-low risk-not amenable to test-unsure (*n* = 1, 2%),strong belief-high risk amenable to test-rejection (*n* = 1, 2%), strong belief-high risk-not amenable to test-rejection (*n* = 1, 2%), strong belief-high risk-not amenable to test-situational acceptance (*n* = 1, 5%), weak belief-high risk-amenable to test-acceptance (*n* = 1, 2%), and weak belief-high risk-amenable to test-situational acceptance (*n* = 1, 2%).

Among 42 individuals (48% > 55 years, 71% female, 55% White/non-Hispanic, 14% Hispanic), most people (*n* = 28, 67%) strongly believed their penicillin allergy was true but had a low-risk allergy history (*n* = 35, 83%). Most people were amenable to penicillin allergy testing (*n* = 39, 93%) and would accept taking penicillin unconditionally (*n* = 20, 48%) or situationally (*n* = 12, 29%) after a negative test result (Fig. 1). All participants with a weak belief of their allergy (*n* = 14, 33%) would be amenable to penicillin allergy testing and would take penicillin unconditionally (*n* = 11, 79%) or situationally (*n* = 3, 21%) after a negative test. No participants with a strong allergy belief and high-risk history (*n* = 5, 12%) reported unconditional acceptance of taking penicillin after a negative test but were generally amenable to penicillin allergy testing (*n* = 3, 60%).

## DISCUSSION

Contrary to clinician concerns, most people with a penicillin allergy were willing to receive penicillin allergy testing and subsequently take penicillin after a negative test result. The level of assessed risk or severity of penicillin allergy history by symptoms differs from individual’s level of belief in the accuracy of their penicillin allergy. These findings contrast clinicians’ impressions about the willingness of patients to discuss their penicillin allergy. Additionally, all participants with a weak allergy belief were willing to get tested and take penicillin unconditionally or situationally. Using a patient’s belief in their allergy could help clinicians identify patients who are willing to discuss their allergy and would change their health behavior by taking penicillin in the future.

Up to 85% of people with a penicillin allergy history are unaware that penicillin allergy testing exists.^5^ Yet, most patients who receive penicillin allergy testing report positive experiences.^5^ Clinical decision support or shared decision-making tools could support penicillin allergy discussions outside allergy clinics. In this study, about one quarter of people reported that after testing negative for a penicillin allergy, they would be reluctant or unsure to take penicillin. Even after allergy testing, many disproven penicillin allergies remain in patients’ electronic health record, and one prior study demonstrated that one in four patients who are de-labeled do not take penicillin.^6^ Identifying patients that are likely to take penicillin after de-labeling allows for appropriate allocation of resources and the greatest improvement in clinical outcomes.^7^

Classifications created for this study were limited by available qualitative in-depth interview data. We decreased interpretation error through iterative review by independent coders. While the Health Belief Model allows for unique insights into penicillin allergy beliefs, other belief models should be considered.

We found that patients with penicillin allergy labels may be more amenable to discussing and testing than assumed by their clinicians. These findings can inform the development of strategies to improve access to penicillin allergy de-labeling.

## Data Availability

Data availability is not applicable due to qualitative nature of the research.
